# Supplementary material for: A lipophilicity-based energy function for membrane-protein modelling and design
Source: PLoS Comput Biol. 2019 Aug 28;15(8):e1007318. doi: 10.1371/journal.pcbi.1007318 (PMC6736313; doi:10.1371/journal.pcbi.1007318)
Supplement: S1 Table — ref2015_memb is identical to the ref2015 energy function, except for the addition of the mp_ terms, whereas RosettaMP is based on score12[10]. For a detailed explanation on energy terms see ref [25,26,84]. 1 used only in centroid-level sampling. 2 used in centroid-level sampling and full-atom sampling of single spanning proteins. (PDF) [file pcbi.1007318.s001.pdf]

Energy term	weight	
	ref2015_memb	RosettaMembrane
fa_atr	1	0.8
fa_dun	0.7	0.01
fa_elec	1	0.026
fa_intra_rep	0.005	0.004
fa_rep	0.55	0.44
fa_sol	1	0
hbond_bb_sc	1	2.34
hbond_lr_bb	1	1.17
hbond_sc	1	2.2
hbond_sr_bb	1	1.17
omega	0.4	0.5
p_aa_pp	0.6	0.32
pro_close	1.25	1
rama_prepro	0.45	(rama) 0.2
ref	1	1
dslf_fa13	1.25	
dslf_ca_dih		5
dslf_cs_ang		2
dslf_ss_dih		5
dslf_ss_dst		0.5
fa_intra_sol_xover4	1	
yhh_planarity	0.625	
lk_ball_wtd	1	
mp_res_lipo	1	
mp_span_angle	1[1]	
mp_helicality	1[2]	
fa_mpenv		0.3
fa_mpenv_smooth		0.5
fa_mpsolv		0.35
fa_pair		0.49
