# Supplementary material for: A lipophilicity-based energy function for membrane-protein modelling and design
Source: PLoS Comput Biol. 2019 Aug 28;15(8):e1007318. doi: 10.1371/journal.pcbi.1007318 (PMC6736313; doi:10.1371/journal.pcbi.1007318)
Supplement: S2 Table — 1small-xxx-small in sequence. (PDF) [file pcbi.1007318.s002.pdf]

PDB	# subunits	Name	Handedness	small-xxx-small[1]	method
2L2T	2	ErbB4	right	yes	NMR
2JWA	2	ErbB2	right	yes	NMR
2MEU	2	VEGFR2 mutant	parallel	no	NMR
2LZL	2	FGFR3tm	left	yes	NMR
1AFO	2	GpA	right	yes	NMR
2K9Y	2	EphA2	left	yes	NMR
2MK9	2	TLR3	right	no	NMR
2HAC	2	Zeta-Zeta TM dimer	left	no	NMR
2K1K	2	EphA1 at pH=4.3	right	yes	NMR
2J5D	2	BNIP3	right	yes	NMR
2M0B	2	ErbB1	right	yes	NMR
2L9U	2	ErbB3	left	no	NMR
2L34	2	DAP12	left	yes	NMR
2L6W	2	PDGFR beta-TM	left	yes	NMR
2LZ3	2	amyloid precursor protein	right	yes	NMR
2J7A	2	NrfH Cytochrome C Quinol Dehydrogenase	right	yes	X-ray
2MIC	2	p75	right	yes	NMR
2KIX	4	nain of BM2 protein from influe			NMR
3LBW	4	M2 closed state			X-ray
2KYV	5	phospholamban			NMR
