# Supplementary material for: A lipophilicity-based energy function for membrane-protein modelling and design
Source: PLoS Comput Biol. 2019 Aug 28;15(8):e1007318. doi: 10.1371/journal.pcbi.1007318 (PMC6736313; doi:10.1371/journal.pcbi.1007318)
Supplement: S3 Table — (PDF) [file pcbi.1007318.s003.pdf]

sequence position	wild-type identity	mutations identity	Experimental ΔΔG (kcal/mol)	RosettaMembrane	ref2015_memb
10	A	F	3.17	1.55	1.37
10	A	I	3.05	0.86	1.19
10	A	L	3.40	-0.26	3.10
10	A	M	4.29	-1.14	0.72
11	G	A	3.49	3.08	3.58
11	G	F	7.53	-0.19	4.68
11	G	I	7.48	5.52	7.40
11	G	L	8.11	1.31	6.70
11	G	M	7.02	1.94	1.51
11	G	V	6.43	5.26	1.05
14	G	A	2.69	0.81	-0.07
14	G	F	5.96	-2.36	-0.48
14	G	I	5.90	0.73	-0.13
14	G	L	5.95	-0.37	-0.23
14	G	M	6.27	0.12	-2.29
14	G	V	5.39	3.37	-0.16
7	G	A	3.20	4.71	3.30
7	G	F	3.93	2.20	5.15
7	G	I	5.12	5.25	8.03
7	G	L	4.22	7.31	8.20
7	G	M	5.35	4.98	5.30
7	G	V	3.74	8.07	2.42
17	L	V	-2.53	1.79	-0.93
3	L	A	-2.29	1.10	-4.22
15	T	A	2.34	-1.13	1.43
15	T	F	6.12	1.78	4.96
15	T	I	5.82	5.05	1.00
15	T	L	6.02	2.75	7.35
15	T	M	5.33	-1.54	1.63
15	T	V	4.25	4.87	0.64
12	V	A	-2.05	0.27	-1.11
12	V	L	2.27	-2.68	2.38
