# Supplementary material for: A lipophilicity-based energy function for membrane-protein modelling and design
Source: PLoS Comput Biol. 2019 Aug 28;15(8):e1007318. doi: 10.1371/journal.pcbi.1007318 (PMC6736313; doi:10.1371/journal.pcbi.1007318)
Supplement: S4 Table — (PDF) [file pcbi.1007318.s004.pdf]

PDB	Protein Name	# subunits
1FX8	Glycerol Facilitator	Tetramer
1K4C	KcsA Potassium Channel, H+ with Fab	Tetramer
1M0L	Bacteriorhodopsin	Trimer
1OTS	H+/Cl- exchange transporter	Dimer
1U19	Rhodopsin (bovine outer segment)	Monomer
2C3E	Mitochondrial ADP/ATP Carrier	Monomer
2UUI	(Apo) Leukotriene Synthase	Trimer
2VPZ	Polysulfide Reductase	Dimer
2XOV	Rhomboid-Family intramembrane protease	Monomer
3B9W	Rh50 protein	Trimer
3GIA	(Apo) ApcT Na+-independent Amino Acid Transporter	Monomer
3K3F	Urea Transporter	Trimer
3KLY	FocA formate transporter w/o formate	Pentamer
3M71	SLAC1 anion channel TehA homolog	Trimer
3O0R	Nitric Oxide Reductase subunit B	Monomer
3RLB	ThiT, S component of the Thiamin Transporter	Dimer
3V5U	Sodium Calcium Exchanger (MCX)	Monomer
3ZOJ	AQY1 Yeast Aquaporin	Tetramer
4A2N	Isoprenylcysteine carboxyl methyltransferase	Monomer
4IKV	Proton-dependent oligopeptide transporter	Monomer
