# Supplementary figures and images for: A lipophilicity-based energy function for membrane-protein modelling and design
Source: PLoS Comput Biol. 2019 Aug 28;15(8):e1007318. doi: 10.1371/journal.pcbi.1007318 (PMC6736313; doi:10.1371/journal.pcbi.1007318)

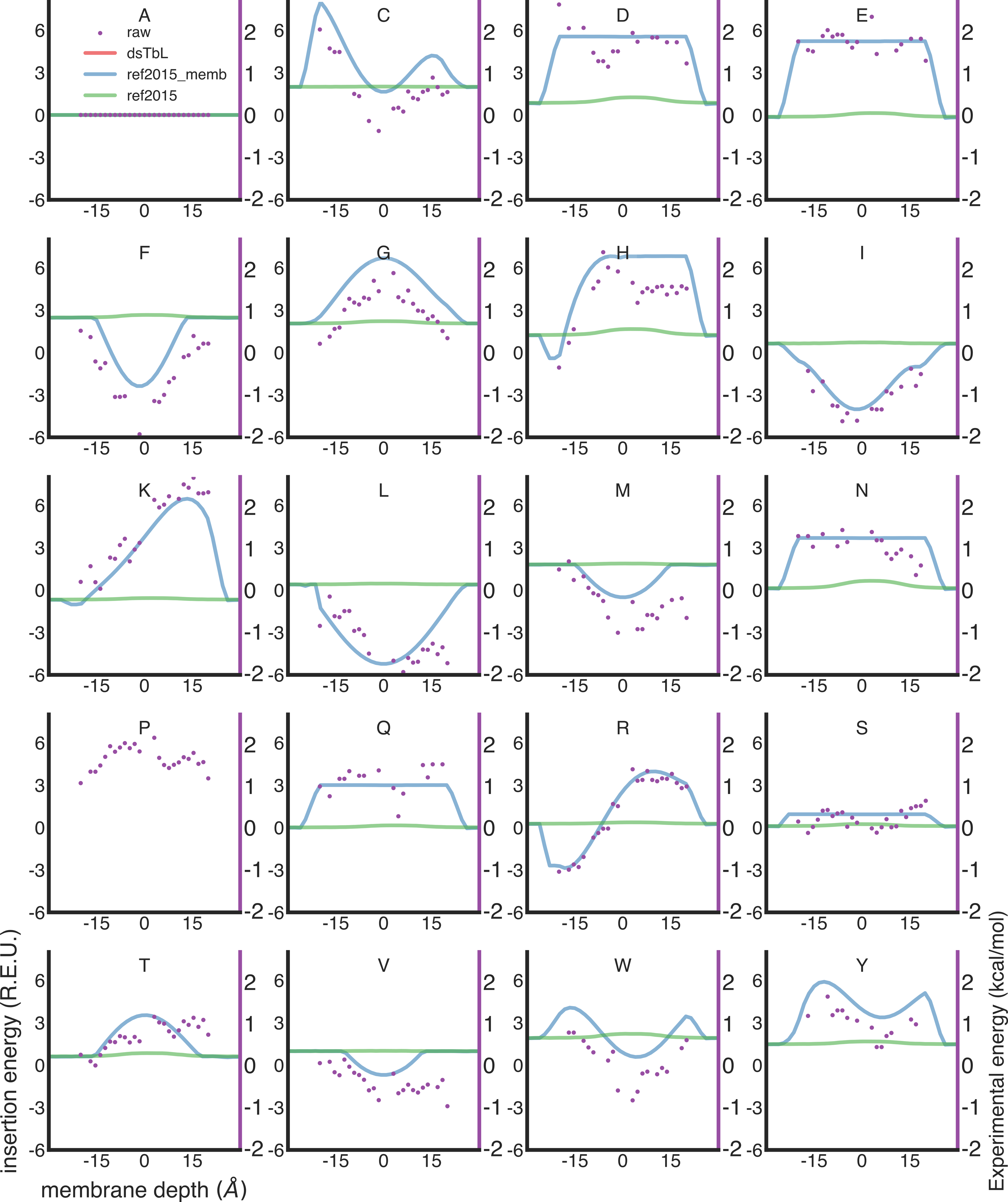

Supplement: S1 Fig — Each panel shows membrane insertion profiles for a different amino acid. Raw dsTβL data (purple dots, right-hand Y-axis), dsTβL adjusted profiles (red line), ref2015 insertion profiles (green) and ref2015_memb profiles (blue dashed line). Different residues affect the α helix differently, and therefore have different baselines. Note that Pro has no profile under ref2015_memb due to its effect on the backbone. (TIF) [file pcbi.1007318.s006.tif]
